# Immunological Monitoring During Anti-CD20 Therapies to Predict Infection Risk and Treatment Response in Multiple Sclerosis Patients

**DOI:** 10.3390/diseases13120387

**Published:** 2025-11-28

**Authors:** Gabriel Torres Iglesias, Ana Martínez-Feito, Laura Otero-Ortega, MariPaz López-Molina, Inmaculada Puertas, Andrea Gonzalez-Torbay, Claudia Geraldine Rita, Mireya Fernández-Fournier, Sara Sánchez Velasco, Beatriz Chamorro, Exuperio Díez-Tejedor, Eduardo López-Granados

**Affiliations:** 1 Neurological Sciences and Cerebrovascular Research Laboratory, Department of Neurology, Neurology and Cerebrovascular Disease Group, Neuroscience Area of Hospital La Paz Institute for Health Research IdiPAZ, La Paz University Hospital, Universidad Autónoma de Madrid, 28046 Madrid, Spain; gabriel.torres.iglesias@salud.madrid.org (G.T.I.); oteroortega.l@gmail.com (L.O.-O.); mplm1995@gmail.com (M.L.-M.); inmaculada.puertas@salud.madrid.org (I.P.); fernandezfournier@hotmail.com (M.F.-F.); sarasanchezvelasco@gmail.com (S.S.V.); beatriz.lapaz2@gmail.com (B.C.); exuperio.diez@salud.madrid.org (E.D.-T.); 2 Clinical Immunology Department, La Paz University Hospital, 28046 Madrid, Spain; amartinezf@salud.madrid.org (A.M.-F.); torbayandrea@gmail.com (A.G.-T.);; 3 Centre for Biomedical Network Research on Rare Diseases (CIBERE U767), 28029 Madrid, Spain; 4 Lymphocyte Pathophysiology in Immunodeficiencies Group, La Paz Institute for Health Research (IdiPAZ), 28046 Madrid, Spain

**Keywords:** multiple sclerosis, immunological monitoring, anti-CD20, infection, immunophenotype

## Abstract

Background: Immunological monitoring in multiple sclerosis (MS) patients treated with disease-modifying drugs may help predict infectious complications and guide treatment. The main objective of this study was to evaluate whether anti-CD20 treatments in MS patients induce immunodeficiency and whether certain immunological parameters can predict the risk of infection and response to treatment. Methods: This retrospective, observational, single-centre study included MS patients who started treatment with ocrelizumab or rituximab and received follow-up in the Neuroimmunology Unit of our centre between January 2017 and January 2023. The study was conducted in collaboration with the Immunology Department of this hospital. Results: Fifty-five patients were included, with a mean age of 47 years and a follow-up period of 24 months. Analyses of lymphocyte subpopulations (T, B, NK) and immunoglobulin levels (IgG, IgA, IgM) were performed before treatment and at 6-, 12- and 24-month follow-ups. In addition, we carried out an exhaustive study of B cells in the baseline analysis. Sixty-four percent of patients presented infections, mostly due to COVID-19. Three patients developed cryptogenic organising pneumonia. IgG hypogammaglobulinemia was the main risk factor for developing infections. Patients with infections had fewer mature memory B cells and a lower percentage of NK cells. Furthermore, a lower proportion of naïve and mature memory B cells was associated with inflammatory activity and disease progression, respectively. The absence of CD20 depletion during follow-up was associated with clinical worsening. Conclusions: Baseline immunophenotype and immunological monitoring can help predict the risk of infections and the efficacy of anti-CD20 therapy in MS patients.

## 1. Introduction

Multiple sclerosis (MS) is the most common chronic immune-mediated disease affecting the central nervous system (CNS) [1]. It is estimated that 2.8 million people worldwide suffer from MS [2], with a prevalence of 100 to 200 cases per 100,000 inhabitants in Europe [3,4]. Symptoms typically begin between the ages of 25 and 30, with MS being the most common non-traumatic neurological disability cause in young adults [5]. Its exact cause, and whether it varies between patients, is still unknown. However, it is believed to arise from the interaction between genetic susceptibility and environmental exposure, leading to a loss of tolerance against CNS antigens, primarily myelin antigens [6]. As a consequence, autoreactive cells are activated and migrate to the CNS through the blood–brain barrier, initiating an immune-mediated response in the CNS. This results in inflammation and demyelination as the main pathological mechanisms, ultimately leading to axonal degeneration and neuronal death. Clinically, MS is characterised by episodes of neurological deficits, typically lasting from days to weeks, which are either fully or partially reversible; this form is known as relapsing-remitting MS (RRMS). Approximately 50% of individuals with RRMS eventually develop a progressive worsening of disability, referred to as secondary progressive MS (SPMS), while about 15% of patients experience continuous progression from disease onset, known as primary progressive MS (PPMS) [7].

Historically considered a CD4^+^ “helper” T-cell mediated disease, recent years have seen increased interest and research into the role of B cells in the pathophysiology of MS. Currently, an immunological landscape has been recognised where there is a complex interaction between B and T lymphocytes, with innate immunity also playing a critical role [8]. This paradigm shift was primarily driven by the success of clinical trials involving selective B-cell depleting therapies with anti-CD20 agents, particularly ocrelizumab (OCR), in patients with RRMS [9] and also with PPMS [10]. At present, OCR is the only disease-modifying treatment (DMT) approved for this form of MS, which has opened new therapeutic possibilities for reducing disease progression in a condition that previously had very limited treatment options. Another anti-CD20 therapy available is rituximab (RTX), which has shown good results in controlling inflammatory activity, although its effects on disease progression remain controversial [11]. Anti-CD20 therapies, in addition to playing a role in preventing progression, are considered among the most effective DMTs for controlling inflammatory activity in MS [12,13].

The main risk associated with chronic immunotherapy based on circulating CD20^+^ B-cell depletion is infectious complications, related to the impairment of antibody-mediated responses and other B-cell functions, such as antigen presentation or cytokine production. While the rates of severe infectious events are not significantly different from those observed in control or placebo groups, serious infections, including opportunistic infections, have been reported. In addition, current clinical trials are often considered to be too short and include sample sizes that are too small to detect such infrequent long-term risks [14]. A significant lesson regarding the infection risk posed by these drugs was learned during the COVID-19 pandemic, which began in 2020. Several studies have shown that these treatments can significantly affect both the clinical and serological course of SARS-CoV-2 infection, as well as long-term immunity and vaccine responses [15], leading to a greater risk of severe infections [16].

The growing availability of DMTs for MS and their potential infectious complications has prompted efforts to individualise treatment schedules and monitoring strategies for each drug based on its characteristics. Since anti-CD20 therapies target a specific immune cell-the B lymphocyte, a key component of humoral immunity-close immunological monitoring can be assessed on an individual basis, allowing personalised treatment. This approach would enable clinicians to stratify patients according to their risk of developing infections, with hypogammaglobulinemia being the main predictor [17,18]. To reduce the risk of infection, patients identified as high risk could benefit from early interventions, such as vaccination, antibiotic prophylaxis, immunoglobulin replacement therapy and adjusting dosage intervals [19].

Immunological monitoring may also serve as a valuable predictive tool for assessing the efficacy of anti-CD20 therapies in patients with MS. This approach has already shown promise in the context of other immune-mediated diseases, where B-cell subpopulation analyses have been used to guide treatment decisions. In MS specifically, some studies have reported that a baseline CD19^+^-cell percentage greater than 12% is associated with insufficient depletion of these cells in the months following treatment, an indicator of reduced therapeutic efficacy [20]. Therefore, baseline immunological studies performed before treatment could help identify previously silent immunological variables and lead to therapeutic adjustments. However, to date, there are no adequately standardised immunological monitoring protocols, and their heterogeneity hinders direct comparisons between studies [21].

Based on the above, we conducted an observational study using an immune monitoring protocol, with the primary goal of evaluating whether OCR and RTX induce secondary immunodeficiency in MS patients and if this is associated with a higher risk of infection, and of assessing the potential role of immunological monitoring as a predictor of both infection and treatment response.

## 2. Materials and Methods

### 2.1. Study Design

This was a retrospective, observational, single-centre study that included patients aged ≥ 18 years diagnosed with MS, according to McDonald’s criteria [6]. They received follow-up in the Neuroimmunology Unit of the Neurology Service at the La Paz University Hospital (Madrid, Spain) between January 2017 and January 2023. The study was conducted in collaboration with the Immunology Department of this hospital, which designed a monitoring protocol and followed patients’ progress. All patients received treatment with OCR or RTX. Exclusion criteria included pregnancy, breastfeeding, substance (drug or alcohol) dependence, serious concomitant disease and participation in clinical trials.

The initial dose of OCR was 600 mg administered as two separate intravenous infusions: a first infusion of 300 mg, followed by a second of 300 mg two weeks later. Subsequent doses were given as a single 600 mg infusion every six months. RTX was administered intravenously at doses of 500–1000 mg every 6–8 months, individualised according to each patient’s clinical status. An initial baseline visit and follow-up assessments every six months were conducted over 24 months, during which clinical data regarding new or recurrent neurological symptoms, infections and laboratory tests were collected, as detailed below.

A pre-treatment evaluation was carried out by the Immunology Department for clinical assessment and baseline immunological testing. Follow-up immunological monitoring was performed prior to each subsequent infusion at 6, 12, 18 and 24 months. After 24 months of treatment, an additional consultation was scheduled to evaluate each patient’s immunological status.

### 2.2. Demographic and Clinical Data

At baseline, the following demographic and clinical data were collected: sex, age, MS subtype (RRMS, PPMS, SPMS), time from MS diagnosis to treatment initiation, Expanded Disability Status Scale (EDSS) score, the reason for switching to anti-CD20 therapy and prior DMTs.

We use the term “fingerprint therapy” (FPT) to encompass DMTs that are considered to have long-term immunological effects. These DMTs included cladribine, natalizumab and alemtuzumab. Currently, MS patients receive several drugs throughout their lives, and these influence the immune system and may leave a long-term mark. Some of the phenomena most closely associated with this are greater likelihood of developing progressive multifocal leukoencephalopathy in patients who have previously received natalizumab, persistent lymphopenias associated with cladribine, and long-term autoimmune events related to alemtuzumab. 

### 2.3. Treatment Safety

Data on infections were collected, distinguishing between COVID-19-related and non-COVID-19 infections. A severe infection was defined as one requiring hospitalisation. Information on potential development of neoplasms and other autoimmune complications was also collected.

### 2.4. Treatment Response

Treatment response was defined using specific clinical variables established by consensus among neurologists on the objectives for monitoring response to DMT [22].

I. Relapses: defined as new or recurrent neurological symptoms not associated with fever lasting ≥ 24 h and followed by at least 30 days of stability or improvement.
II. Brain magnetic resonance imaging (MRI) activity: defined as the presence of at least one new or enlarged lesion in a T2-weighted MRI scan at 12 months. An annual brain MRI was performed for all patients to detect radiological activity.
III. Disease progression: defined as an increase of 1.5 points in the EDSS score if the baseline EDSS score was 0, 1.0 point if baseline EDSS was between 1 and 5.5, and 0.5 points if baseline EDSS was ≥6.0 [23].

All clinical evaluations and outcome measurements were performed exclusively by experienced neurologists. Treatment response was assessed according to the guidelines of the European Committee for Treatment and Research in Multiple Sclerosis (ECTRIMS) and the NEDA-3 (No Evidence of Disease Activity-3) criteria, defined as the absence of relapses, MRI activity and disability progression [24]. Inflammatory activity was defined as disease activity manifested by relapses and/or new CNS lesions on MRI [25].

### 2.5. Immunological Studies

At each visit, peripheral blood samples were collected in EDTA tubes. At the initial visit, the baseline immunophenotype of patients was analysed, recording data such as CD4^+^ T cells, CD8^+^ T cells, CD4^+^/CD8^+^ ratio, B CD19^+^, NK CD56^+^ and CD12^+^ cells, and a more detailed characterisation of the B-cell compartment. Patients with a CD19^+^ percentage > 12% were also specifically analysed. Humoral parameters, including immunoglobulin levels (IgG, IgM, IgA) were also recorded.

Flow cytometry (DxFlex, Beckman Coulter) was used to identify the following lymphocyte subpopulations: T (CD3^+^CD4^+^, CD3^+^CD8^+^), B (CD19^+^), and NK (CD16^+^/56^+^), with a detailed analysis of B-cell subpopulations, including: naïve (CD19^+^, IgD^-^, CD27^-^), unswitched memory (CD19^+^, IgD^+^, IgM^+^ CD27^+^), switched memory (CD19^+^, IgD^-^, IgM^-^, CD27^-^), transitional (CD19^+^, IgM^+^, CD38^+^), plasmablast (CD19^+^, CD38^+^, IgM^low^), and CD21^low^ B cells (CD19^+^, CD21^low^, CD38^+^). Immunoglobulin levels (mg/L) were determined by nephelometry.

During follow-up visits at 6, 12, 18 and 24 months, immunological monitoring included collection of the following data: absolute counts and percentages of T-, B- and NK-cell population, as well as serum IgG, IgM, IgA levels.

Cases of lymphopenia were recorded and classified as follows: grade 1: lymphocytes 999–800/µL; grade 2: lymphocytes 799–500/µL; grade 3: lymphocytes 499–200/µL; grade 4: lymphocytes < 199/µL [26]. Hypogammaglobulinemia was defined as immunoglobulin levels below the following thresholds: IgG < 700 mg/dL; IgM < 45 mg/dL; IgA < 50 mg/dL [27]. Reference values for different cell subtypes were taken from Schatorjé et al. [28] and for memory B-cell subtypes from Wehr et al. [29].

Immunological abnormalities were considered persistent if they were present in ≥50% of the follow-up assessments (lymphopenia, hypogammaglobulinemia, CD4^+^/CD8^+^ ratio). Progressive depletion of B cells due to treatment was also evaluated by measuring absolute numbers and percentages of CD19^+^ cells.

### 2.6. Statistical Analysis

Categorical variables were summarised as percentages, while continuous variables were expressed as means with standard deviations (SD) or medians with interquartile ranges (IQR), as appropriate. Changes in laboratory variables across successive follow-up visits were analysed by comparing mean values. A univariable analysis using contingency tables was performed to explore the relationships between qualitative variables, and a multivariable analysis was subsequently applied for variables that showed significance (*p* < 0.1) in the univariable model. Statistical analyses were performed using Student’s t-test for continuous variables and Chi-square or Fisher’s exact test, as appropriate, for categorical variables. The Wilcoxon signed-test was used when normality assumptions were not met. Non-normally distributed data were compared using the Kruskal–Wallis or the Mann–Whitney U test. Binary logistic regression and ROC curve analyses were used to identify cell count thresholds predictive of infection or treatment response. To assess differences during follow-up of immunological subpopulations and immunoglobulins, we used the ordinary one-way ANOVA test. Multiple comparisons were adjusted using Bonferroni correction. Results with an alpha level < 0.05 were considered statistically significant. All analyses were performed using SPSS version 26.0 for Windows (SPSS Inc., Chicago, IL, USA).

## 3. Results

### 3.1. Clinical and Demographic Characteristics of the Patients

Fifty-five patients were included in the study, of whom 40 were treated with OCR and 15 with RTX. The follow-up period lasted 24 months. The median age of the patients was 46.2 years, with more than half being women (54.5%). Most patients had RRMS (49%) and in most cases anti-CD20 drugs were not the first treatment they had received (81.5%). Other patient characteristics are summarised in Table 1.

### 3.2. Immunological Study

#### 3.2.1. Baseline Immunophenotype

Table 2 shows the cell subpopulations and immunoglobulin levels derived from the patients baseline immunophenotype.

In the baseline immunological study, 26.6% of patients showed a percentage of CD19^+^ cells over 12%.

**Figure 1:**
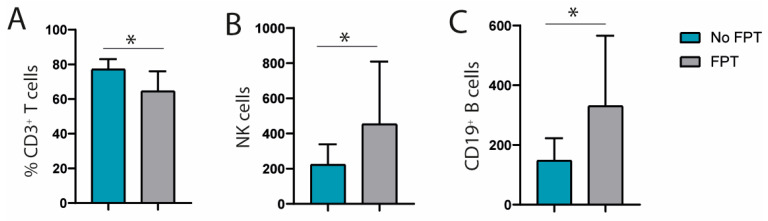
Differences in baseline immunophenotype (T, B and NK cells) between patients with or without prior FPT. (**A**) Percentage CD3^+^ cells in patients with prior FPT. (**B**) Number of NK cells in patients with prior FPT. (**C**) Number of CD19^+^ cells in patients with prior FPT. * Indicates groups with significant differences.

We compared the different lymphocyte subpopulations between patients who had previously received FPT and those who had not. We found a lower percentage of CD3^+^ T cells in patients with prior FPT (64.53 ± 11.53% vs. 77.15 ± 5.92%; *p* = 0.031). In addition, patients with prior FPT had higher absolute NK-cell counts (453.17 ± 356.23 cells/μL vs. 223.18 ± 115.62 cells/μL; *p* = 0.031), and higher absolute CD19^+^ B-cell counts (330.3 ± 235.83 cells/μL and 147 ± 75.58 cells/μL; *p* = 0.027) (Figure 1).

In 28 (51%) patients, an exhaustive analysis of the B compartment was performed; however, no significant differences were found between patients with or without prior FPT (Supplementary Table S1).

#### 3.2.2. Immunological Monitoring

CD4^+^, CD8^+^ and NK-cell monitoring and development of persistent lymphopenia.

**Figure 2:**
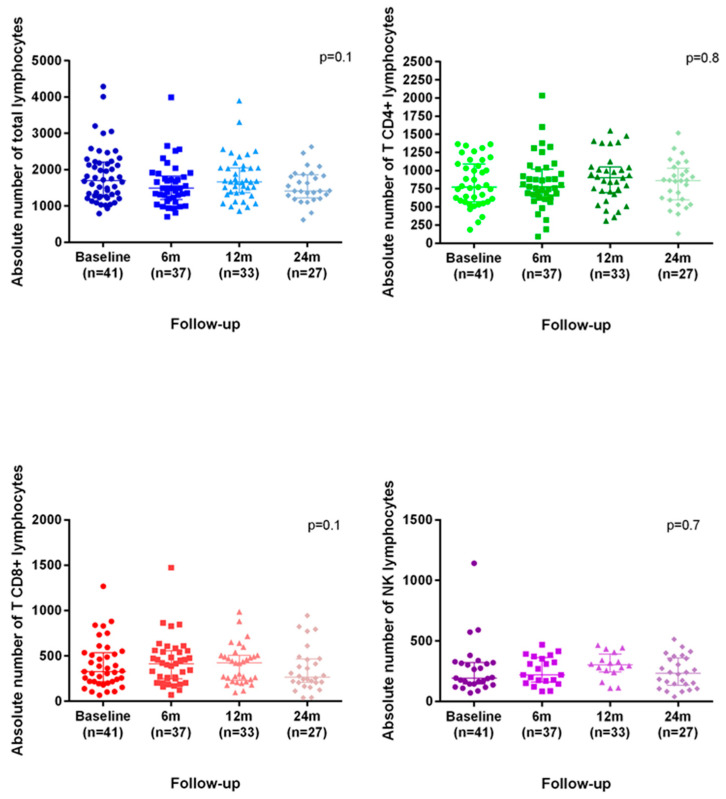
Monitoring of CD4, CD8 and NK cells.

Immunological data were recorded every six months from the start of treatment. The CD4^+^ and NK cell subpopulations did not show a significant decrease during follow-up. However, CD8^+^ cells showed a downward trend, which was not statistically significant (Figure 2 and Table 3). Only 3 patients (6%) out of 55 presented a persistently inverted CD4^+^/CD8^+^ ratio (Table 4). Five patients (8.9%) developed persistent lymphopenia, all of which were mild (grade 1) (Table 4). Four patients had CD4^+^ levels below 200 in a single isolated measurement.

Decrease in B cells.

**Figure 3:**
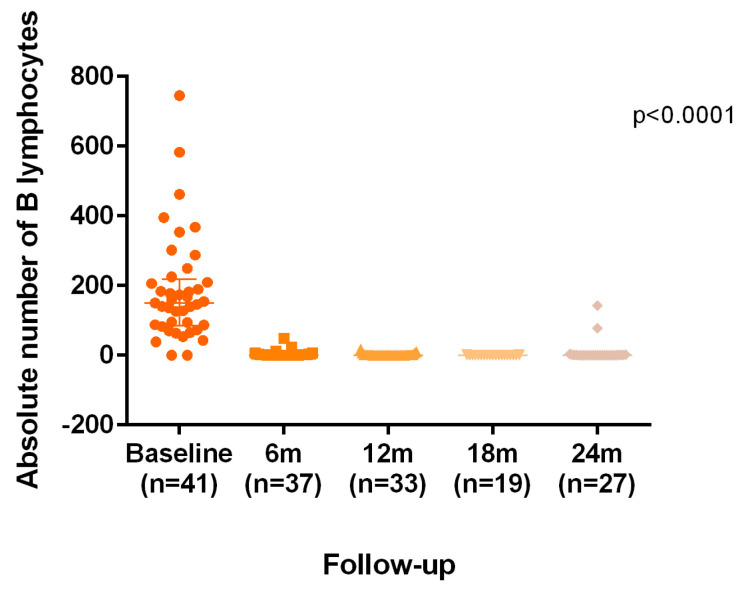
B-cell monitoring.

From the first infusion of both anti-CD20 therapies, a marked decrease in CD19^+^ cell counts was observed, with a mean of 10 ± 23 cells/μL at 6 months and 7 ± 26 cells/μL at 12 months. At 6 months, 51.3% of patients who underwent cell subpopulation analysis showed complete depletion of CD19^+^ cells, with 53.8% in the OCR group and 45.5% in the RTX group (Table 4). At 12 months, 60.6% of patients showed complete absence of CD19^+^ cells, with 61.5% in the OCR group and 57.1% in the RTX group (Table 4). After 12 months of follow-up, virtually all patients exhibited 0 CD19^+^ cells. The progressive decline in B cells is illustrated in Figure 3.

Humoral compartment

**Figure 4:**
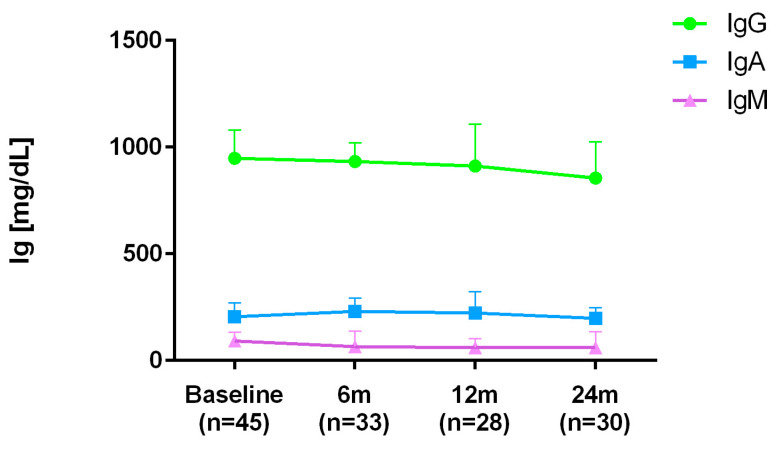
Immunoglobulin monitoring.

A gradual decrease in IgG levels was observed throughout the follow-up, although it did not reach statistical significance (Table 3 and Figure 4). Eighteen percent of patients had persistently low IgG levels below normal limits (Table 4).

Comparatively, IgM levels showed a marked downward trend throughout the follow-up period (Table 3 and Figure 4). Twenty-one percent of patients had persistently low IgM levels below the normal range (Table 4).

### 3.3. Safety: Development of Infections and Neoplasms

Thirty-five (63.6%) patients experienced infections during the study, 22 (63%) of which were related to COVID-19 and 13 (37%) were non-COVID-19 infections. Among the non-COVID-19 infections, 53.7% were bacterial urinary tract infections and 30.7% were other viral respiratory infections. Three of the COVID-19 infected patients had a severe clinical course, complicated by cryptogenic organised pneumonia (COP). One patient developed febrile neutropenia (Table 5). No cases of tuberculosis were observed during anti-CD20 therapy in our study.

Our analysis of the COVID-19 vaccination status of the patients showed that all but three (5.4%) were vaccinated against COVID-19, and all received the vaccine in 2021, with June 2021 being both the median and most frequent month of vaccination. Among the 52 vaccinated patients, 22 (42.3%) contracted COVID-19 and 25 (53.2%) did not. Only two patients (3.8%) developed COVID-19 infection before vaccination. The four patients who experienced severe COVID-19 infection had all been vaccinated prior to infection.

**Figure 5:**
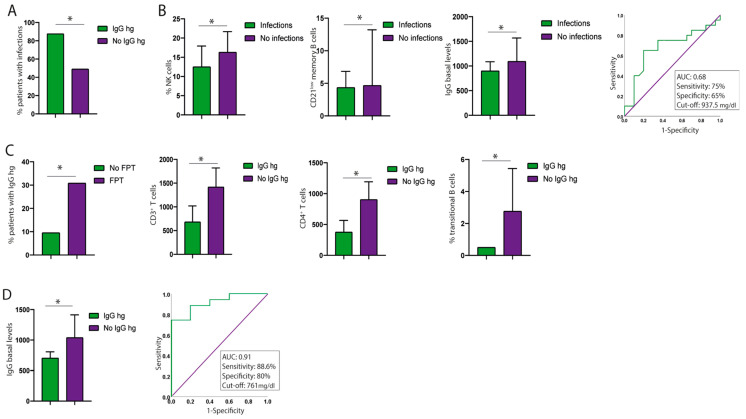
Association of immunophenotype profiles with infection and persistent hypogammaglobulinemia (hg). (**A**) Baseline hypogammaglobulinemia and infections. (**B**) Percentage NK cells, absolute CD21^low^ B-cell counts and IgG baseline levels and infection. (**C**) FPT patients, baseline absolute CD3^+^ T-cell counts, baseline absolute CD4^+^ T-cell counts, baseline percentage of transitional B-cells and persistent hypogammaglobulinemia. (**D**) Baseline IgG levels and development of persistent hypogammaglobulinemia. * Indicates groups with significant differences.

Regarding infection risk factors, the development of IgG hypogammaglobulinemia was significantly associated with infections (*p* = 0.043, CI [1.208–2.647]) (Table 6, Figure 5A). This association was not observed for other immunological variables such as persistent IgM hypogammaglobulinemia or persistent lymphopenia. Likewise, no significant associations were found with age, sex, progressive forms of MS, EDSS score, or prior FPT (Table 6).

Given that the high incidence of COVID-19 infection could have influenced the results, we conducted a secondary analysis to evaluate the association between non-COVID-19 infections and persistent IgG hypogammaglobulinemia. This analysis also demonstrated a statistically significant association (*p* = 0.045; CI, 2–70%)

When analysing the baseline immunophenotype, patients who developed any infection (COVID-19 or non-COVID-19) had a lower absolute number of CD21^low^ memory B cells (4.33 ± 2.51 cells/μL and 4.66 ± 8.56 cells/μL; *p* = 0.049), a lower percentage of NK cells (12.5 ± 5.43 cells/μL and 16.3 ± 5.37 cells/μL; *p* = 0.045), and lower IgG levels (899.35 ± 188.77 mg/dL and 1092.75 ± 474.36 mg/dL; *p* = 0.048). A baseline IgG cutoff of 937.5 mg/dL was associated with the development of infections, with 75% sensitivity and 65% specificity (AUC = 0.68, CI [0.51–0.85]) (Figure 5B).

Regarding the development of persistent IgG hypogammaglobulinemia, significant associations were found with: prior FPT (*p* = 0.048, CI [1.053–−6.477]), lower absolute CD3^+^ T-cell counts (680 ± 339.41 cells/μL and 1417.62 ± 402.56 cells/μL; *p* = 0.04), lower absolute CD4^+^ T-cell counts (375 ± 190.92 cells/μL and 903.19 ± 288.28 cells/μL; *p* = 0.026), lower percentages of transitional B cells (0.50 ± 0.01 cells/μL and 2.76 ± 2.67 cells/μL; *p* = 0.032) at baseline, and reduced baseline IgG levels (703 ± 103.25 mg/dL and 1037.91 ± 375.46 mg/dL; *p* = 0.03) (Figure 5C,D). Furthermore, a baseline IgG cutoff of 761 mg/dL was associated with the development of persistent hypogammaglobulinemia with 88.6% sensitivity and 80% specificity (AUC = 0.91, CI [0.81–1.00]) (Figure 5D). No associations were observed between the development of IgG hypogammaglobulinemia and age or sex.

All patients with a single CD4^+^ determination < 200 presented infection during follow-up, none of which was serious.

No new neoplasms were detected in any patient.

### 3.4. Efficacy: Activity, Progression and NEDA-3

A total of 89.1% of patients showed no signs of inflammatory activity. Among those who did, 9.1% exhibited new lesions on MRI and 3.6% experienced new relapses. Based on EDSS criteria, 30.9% of patients met criteria for disease progression. In terms of NEDA-3, 61.8% of patients remained free of disease activity (Table 7).

**Figure 6:**
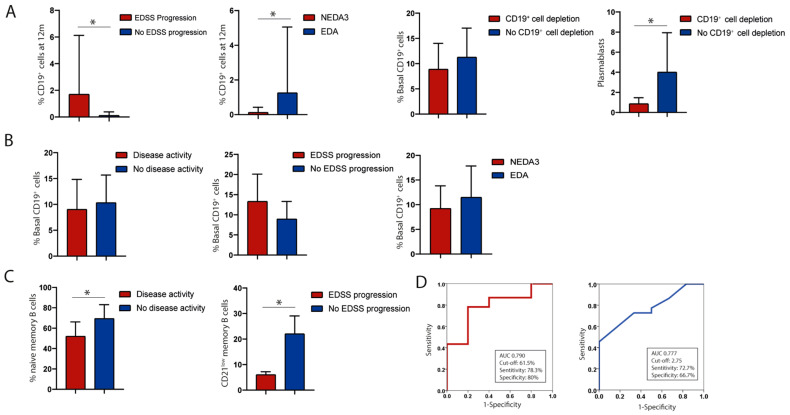
Association between baseline total CD19^+^ and treatment efficacy. (**A**) Baseline plasmablast and B-cell depletion at 12 months. (**B**) Baseline percentage of CD19^+^ cell and treatment efficacy. (**C**) and (**D**) Percentage of naïve B cells and disease activity; absolute CD21^low^ B-cell numbers and disease progression. * Indicates groups with significant differences.

The relationship between anti-CD20 therapy efficacy and lack of B-cell depletion in follow-up analyses was also assessed. At the 12-month follow-up, patients who did not achieve B-cell depletion showed disease progression (1.71% ± 4.43 vs. 0.11% ± 0.28, *p* = 0.047) and did not meet NEDA-3 criteria (1.25% ± 0.30 vs. 0.12% ± 0.30, *p* = 0.049) (Figure 6A). However, no significant relationship was observed between the baseline percentage of total B cell and the lack of depletion at 12 months (*p* = 0.64, CI [−12.29–7.78]).

When analysing different B-cell subpopulations at baseline, a higher percentage and absolute number of plasmablasts were associated with incomplete B-cell depletion at 12 months (0.86 ± 0.63 cells/μL vs. 4.0 ± 3.937 cells/μL, *p* = 0.04) (Figure 6A). The potential association between baseline CD19^+^ percentage and treatment efficacy was also analysed, but no significant correlation was found (*p* = 0.27, CI [−2.44–8.27]) (Figure 6B). Likewise, no association was observed between a CD19^+^ cell percentage greater than 12% at baseline and treatment efficacy. However, when evaluating specific CD19^+^ subpopulations at the first pre-treatment visit, significant associations emerged. A lower percentage of naïve B cells (CD19^+^, IgD^-^, CD27) was associated with inflammatory activity (51.98 ± 14.23%) compared to patients with stable disease (69.4 ± 13.74%, *p* = 0.045). Additionally, a lower absolute number of CD21^low^ B cells was associated with disease progression (6 ± 1.2 cells/μL) compared to those with stable disease (22 ± 7.04 cells/μL, *p* = 0.039) (Figure 6C). A ROC curve analysis identified a cutoff of 61.5% naïve B cells predictive of inflammatory activity (78.3% sensitivity and 80% specificity, AUC = 0.79, CI [0.59–0.99]), and a cutoff of 2.75 CD21^low^ B cells predictive of disease progression (72.7% sensitivity and 66.7% specificity, AUC = 0.78, CI [0.60–0.96]) (Figure 6D).

No significant differences in efficacy variables were observed between patients who had received FPT and those who had not (*p* = 0.48, CI [22–85%]).

## 4. Discussion

Our study highlights the importance of immune monitoring during anti-CD20 therapy in patients with MS. More than half of the patients experienced infections during follow-up; however, most were mild. A notable exception was the development of COP following COVID-19 infection, which warrants further investigation. Overall, anti-CD20 therapies showed high efficacy in controlling inflammatory disease activity; however, their impact on disease progression remains partial, and alternative therapeutic strategies for this purpose are currently lacking. Persistent IgG hypogammaglobulinemia was identified as the main risk factor for developing infection. Moreover, the absence of CD19^+^ B-cell depletion at 12 months after treatment was found to be the primary risk factor associated with therapeutic failure. Notably, the most novel contribution of our study was the association between baseline immunophenotype and both infection risk and treatment efficacy, underscoring the potential value of immunological profiling in guiding personalised therapeutic strategies.

One of the main objectives of this study was to evaluate the safety profile of anti-CD20 therapies. Thirty-five patients developed infections, 22 suffered COVID-19 (severe in 4 cases) and 13 had non-COVID infections, the most frequent being urinary and respiratory tract infections. Most infections were mild and did not cause major complications. In clinical trials of OCR in MS, no higher rate of serious infections was observed compared with interferon in RRMS and placebo in PPMS [9,10]. However, both studies reported higher rates of upper respiratory tract and herpesvirus infections. Real-world studies have shown higher infection rates compared with open-label extension studies of anti-CD20 therapies, particularly upper respiratory and urinary tract infections, although the rate of serious infections remains low [30,31].

COVID-19 was the most common infection among patients, likely reflecting the time period in which the study was conducted. However, almost all patients had been vaccinated, and among those who contracted COVID-19, all but two were infected after vaccination. Furthermore, of the 22 patients who presented COVID infection, 4 of them were severe. For this reason, although the epidemiological context explains the high frequency of COVID-19 infections, the incidence of both COVID-19 and severe COVID-19 was higher than expected for the general population. Several studies have demonstrated an association between anti-CD20 therapy and an increased likelihood of a more severe COVID-19 clinical course in MS patients [32,33]. There is growing evidence of a diminished humoral response to SARS-CoV-2 infection in MS patients treated with OCR [34], as well as an attenuated humoral immune response to the COVID-19 vaccine in those treated with anti-CD20 agents [15]. In contrast, T-cell responses appear to be preserved and comparable to those of healthy individuals, even in cases of failed seroconversion [35,36]. Based on these findings, during the COVID-19 pandemic, extended dosing intervals for OCR were proposed as a risk mitigation strategy [37,38].

Only one patient developed bacterial pneumonia requiring hospitalisation (etiological agent not identified). On the other hand, three patients developed COP. This is a complication described in anti-CD20 therapy, especially since the COVID pandemic. Although its pathogenesis is unclear, it is known that COP originates from lung injury, such as COVID infection, as a reparative reaction of the alveolar epithelium with a subsequent inflammatory response. Anti-CD20 therapy reduces the B-cell population and can cause dysregulation of regulatory T cells, initiating an inflammatory response that leads to COP [39]. In our study, no association was found between the occurrence of COP and persistent lymphopenia, hypogammaglobulinemia, prior immunosuppressive therapy or baseline immunophenotype.

Identifying risk factors associated with infection development would allow for personalised management and early preventive strategies. Previous studies have identified several risk factors, including older age, greater neurological disability, longer treatment duration and prior exposure to immunosuppressive agents [17,40]. However, in our cohort, no significant differences associated with these factors were found. Similarly, no differences were found between patients receiving OCR or RTX, despite the latter group having a worse baseline neurological status and a higher frequency of progressive MS. Furthermore, no association was found between infections and lymphopenia. These results indicate that additional immunological parameters may be more relevant in determining susceptibility to infections during anti-CD20 therapy. Consequently, immunological monitoring could provide valuable insight for identifying patients at greater risk. In our assessment of infection risk according to baseline immunophenotype, we found that a lower percentage of NK cells and a lower absolute number of mature memory B cells before treatment were associated with a higher infection rate. It is reasonable to assume that patients receiving anti-CD20 therapy, which broadly reduces circulating B cells, are at greater risk of infection if they already have fewer mature memory B cells at baseline. These cells play a crucial role in protecting against previously encountered pathogens and form the basis of vaccine-induced immunity [41]. NK cells are also essential for protection against infections, particularly viral infections, which were the most frequent in our study [42]. These findings underscore the importance of baseline immunophenotype in identifying patients at higher risk of infectious complications.

Previous studies have found the main risk factor for infection development in MS patients treated with anti-CD20 therapy to be hypogammaglobulinemia [43,44]. During follow-up, we observed a decrease in IgM and IgG from 6 months onward, more pronounced for IgM, although none were significant. Other authors have reported a selective decrease in IgM in longitudinal studies [10,45]. When only circulating B cells are analysed, IgG and IgA levels are expected to remain stable, as long-lived plasma cells in the bone marrow and lymphoid tissues reservoirs continue antibody production. However, in our study, persistent hypogammaglobulinemia associated with infections involved IgG rather than IgM, consistent with clinical trials of OCR [46]. Other studies did not find an association between serum IgG levels and infection risk with OCR [44,47], possibly due to short follow-up periods or underreporting [43,44]. Currently, there is no expert-recommended protocol for immunological monitoring in MS patients treated with anti-CD20 therapy, unlike other autoimmune diseases [48]. Implementing such monitoring in clinical practice could help identify high-risk patients and enable preventive interventions such as antimicrobial prophylaxis, immunoglobulin replacement therapy and adjusting dosage intervals.

Based on the above, many of the studies evaluating the safety of anti-CD20 therapies in MS have analysed potential risk factors for hypogammaglobulinemia development. Regarding the evaluation of these risk factors, some demographic and clinical variables have been associated with its development, such as ages over 50, white ethnicity and RTX use [18]. However, we did not observe any relationship with age or gender. Regarding immunological parameters, lower baseline immunoglobulin levels before starting anti-CD20 therapy and the presence of primary immunodeficiency have been linked to the development of hypogammaglobulinemia [49]. In our study, lower IgG baseline levels were significantly associated with the subsequent development of persistent IgG hypogammaglobulinemia, with a cutoff value of 761 mg/dL. These findings suggest that measuring baseline IgG levels may predict the risk of developing hypogammaglobulinemia during treatment and highlights the need for more detailed baseline immunological monitoring in these patients.

The other immunological risk factors associated with subsequent persistent IgG hypogammaglobulinemia were a lower absolute count of CD3^+^ and CD4^+^ T cells and a lower percentage of transitional B cells in the baseline analysis. The reduction in transitional B cells impairs the host’s ability to maintain antibody-mediated protection against infections [50]. Additionally, in MS, transitional B cells play a role in disease pathogenesis by promoting immune tolerance, and other studies have found these cells to be reduced in MS patients, increasing after the initiation of DMT [51,52]. Moreover, it is biologically plausible that the reduction in CD3^+^ and CD4^+^ T cells at baseline is related to subsequent hypogammaglobulinemia, as CD4^+^ T helper cells are essential for B-cell activation and antibody production after antigen presentation. Few studies have analysed the association between baseline immunophenotype and hypogammaglobulinemia. A review of RTX use in patients with autoimmune and onco-hematologic diseases reported that those with lower baseline IgG levels more frequently presented IgG hypogammaglobulinemia during follow-up [53].

In addition to the safety analysis, we also evaluated the potential role of immunological monitoring as a predictor of treatment response. A high percentage of patients (61.8%) remained in NEDA-3 throughout follow-up, with EDSS progression in 30.9% and very little inflammatory activity (10.9%). These favourable results are comparable to those observed in other cohorts regarding the efficacy of anti-CD20 therapies in clinical practice [54,55], supporting the inclusion of these treatments as highly effective options.

Previous studies have shown a trend toward a reduced risk of NEDA-3 failure in treatment-naïve patients [56]. In our cohort, no statistically significant association was found between treatment efficacy and prior treatments. One factor that may influence the efficacy of anti-CD20 therapies is the baseline immunophenotype of patients previously treated with immunosuppressive agents. We analysed how prior treatments may have modified the baseline immunophenotype of our patients and found that those who had received cladribine, natalizumab or alemtuzumab had a significantly lower percentage of CD3^+^ T cells, higher absolute NK cell counts, and higher absolute CD19^+^ B cell counts. While alemtuzumab causes depletion of all of these cell types, repopulation occurs more rapidly and can reach or even exceed baseline B cell levels, whereas T cell recovery is slower and often remains below baseline [57]. Similarly, cladribine causes selective depletion of B and T cells, but repopulation is faster and more complete for B cells [58]. However, in our cohort, no significant association was found between treatment efficacy and these therapies, perhaps because most patients were switching from other types of drugs that were less effective.

Rapid B cell repopulation is one of the best-known risk factors for anti-CD20 therapy failure in MS [37,59]. This is reasonable, since repopulation of the B cells targeted by therapy is probably related to the re-emergence of immune-mediated pathogenic mechanisms. Similarly, in our immunological follow-up we observed that the presence of CD19^+^ cells at 12 months after treatment was associated with EDSS progression and the absence of NEDA-3. However, studies conducted during the COVID-19 pandemic, in which anti-CD20 dosing intervals were extended and thus B cell repopulation occurred, have not shown reduced treatment efficacy [38]. Following this rationale, in MS treated with RTX, where administration protocols vary between centres, a cutoff point of ≥0.2% B cells in follow-up analyses has recently been proposed as a retreatment threshold, demonstrating high efficacy and a favourable safety profile [60]. Despite the favourable results regarding inflammatory disease activity in our study, control of disease progression remains unsatisfactory. This is likely due to compartmentalised inflammation within the CNS that these drugs cannot effectively target. As mentioned above, the lack of CD19^+^ cell depletion at 12 months was associated with the absence of NEDA-3, primarily due to disease progression. It is possible that complete CD19^+^ cell depletion is necessary to minimise subsequent compartmentalised inflammation, even considering shorter infusion intervals if CD19+ cells begin to reappear.

Higher baseline CD19^+^ cell counts may lead to incomplete CD19^+^ cell depletion suggesting that OCR dose adjustments could be considered in these patients. One study identified a threshold of 12% baseline CD19^+^ cells for B cell repopulation at 6 months and 14% at 12 months, suggesting that these patients could benefit from a higher dose during the first two cycles or a shorter dosing interval before the next infusion [20]. Therefore, establishing an initial predictive immunophenotype-based risk of treatment failure could be highly valuable for personalising therapy. In our study, we found no association between total baseline CD19^+^ B cells and lack of B-cell depletion at 12 months, even when performing a subanalysis of patients with CD19^+^ levels greater than 12% of total cells. However, when analysing specific B cell subpopulations, we observed that higher baseline plasmablasts counts were associated with lack of CD19^+^ cell depletion at 12 months. This may be explained by the fact that plasmablasts are the final B cell stage before plasma cells and are CD19^+^ but CD20^-^, meaning that anti-CD20 therapies have little or no direct effect on them, thereby favouring a higher CD19^+^ cell count at 12 months. Patients with a higher baseline plasmablast component might therefore benefit from anti-CD19 therapies under investigation for MS, such as inebilizumab or even CAR-T cell therapies against CD19.

We also did not find an association between baseline CD19^+^ cells and NEDA-3, disease activity, or progression. However, when analysing baseline B cell subpopulations, we observed that a low percentage of naïve memory B cells was significantly associated with inflammatory disease activity during follow-up, and that a lower absolute number of CD21low memory B cells is significantly associated with disease progression. This may indicate that MS patients with a low memory B cell burden are less dependent on humoral immune mechanisms, and therefore anti-CD20 therapies may be less effective in these individuals. Furthermore, as previously mentioned, since patients with lower baseline levels of mature memory B cells appear to be more susceptible to infections, such patients may not be ideal candidates for anti-CD20 therapies.

This study has several limitations. First, it is retrospective in nature and therefore subject to inherent biases, such as incomplete data for some patients resulting from the lack of a standardised protocol. Moreover, this study reflects real-world clinical practice, which may vary according to the discretion of the treating physician, particularly regarding RTX dosing and administration intervals, potentially introducing variability in the results. There was also a notable imbalance between the OCR and RTX groups, with the former being considerably larger, reflecting current trends in the use of anti-CD20 therapies for MS. To assess whether these differences affected the outcomes, we performed a comparative analysis between the OCR and RTX groups. As COVID infection was the most frequent one, this could have influenced the outcome of the global rate of infections. However, the vast majority of patients were vaccinated, and the infection occurred after the vaccine was administered. Despite this, the frequency of COVID infection was high, including cases of severe COVID, so we consider that anti-CD20 therapy may have influenced these results. Finally, this is an exploratory study, and its findings require validation in a larger, prospective cohort.

## 5. Conclusions

Our findings highlight the critical role of immunological assessment in ensuring the safety and efficacy of anti-CD20 therapies in patients with MS. Baseline and regular monitoring of immune parameters not only helps identify individuals at higher risk of infection but also provides valuable insight into treatment response, enabling more personalised therapeutic strategies. Implementing such immunological monitoring could ultimately improve clinical outcomes, minimise adverse events, and optimise overall care of MS patients receiving anti-CD20 therapy.

## Figures and Tables

**Table 1:** Demographic and clinical characteristics of MS patients treated with anti-CD20 therapies (*n* = 55) and patients treated with OCR (*n* = 40) and RTX (*n* = 15).

	Total (*n* = 55)	OCR (*n* = 40)	RTX (*n* = 15)	*p*-Value
Mean age (SD) years	46.86 (8.5)	44 (6.8)	52 (10.1	0.08
Sex, *n* (%)				0.48
woman	30 (54.5)	23 (57.5)	7 (46.7)	
men	25 (45.5)	17 (42.5)	8 (53.3)	
Time course (previous OCR/RTX)				0.92
mean (SD) years	9.9 (8.7)	9.5 (7.8)	11.3 (11.3)	
Type of MS, *n* (%)				0.007 *
1. RRMS	27 (49)	27 (67.5)		
2. SPMS	20 (36.4)	9 (22.5)	11 (73.3)	
3. PPMS	8 (14.6)	4 (10)	4 (26.7)	
EDSS baseline (median, interquartile)	4.5 (1.5–6)	2.5 (1.5–5)	6 (2.5–6)	0.03 *
Reason for change, *n* (%)				0.31
1. Relapse	5 (9.1)	5 (14.3)	0	
2. New lesions MRI	10 (18.2)	9 (25.7)	1 (6.7)	
3. Clinical progression	12 (21.8)	8 (22.8)	4 (26.7)	
4. Adverse effects	8 (14.5)	4 (11.4)	4 (26.7)	
5. Clinical trial	1 (1.8)	1 (2.8)	0	
6. PML ^ risk	3 (5.5)	2 (5.7)	1 (6.7)	
7. New relapses and lesions	6 (10.9)	6 (17.1)	0	
Pre-treatment OCR/RTX, *n* (%)				0.70
None	10 (18.5)	5 (12.5)	5 (33.3)	
Interferon	8 (14.5)	6 (15)	2 (13.3)	
Azathioprine	3 (5.5)	1 (2.5)	2 (13.3)	
Glatiramer acetate	6 (10.9)	6 (15)	0	
Teriflunomide	7 (12.7)	6 (15)	1 (6.7)	
Dimethyl fumarate	8 (14.2)	7 (17.5)	1 (6.7)	
Fingolimod	2 (3.6)	1 (2.5)	1 (6.7)	
Natalizumab	9 (16.4)	7 (17.5)	2 (13.3)	
30 (75)	10 (20) 30 (75)	1 (2.59)	1 (6.7)	
10 (20) 30 (75)	10 (20) 30 (75)	0	2 (13.3)	
Treatment fingerprint (FPT), *n* (%)				0.70
Yes	13 (24)	10 (20)	3 (20)	
No	42 (76)	30 (75)	12 (80)	
Oncological history, *n* (%)				0.28
Yes	1 (1.8)	0	1 (6.7)	
No	54 (98.2)	55 (100)	14 (93.3)	
Other autoimmune diseases, *n* (%)				0.83
Yes	3 (5.5)	2 (5)	1 (6.7)	
No	52 (94.5)	38 (95)	14 (93.3)	
COPD ^†^, *n* (%)				0.48
Yes	2 (3.6)	1 (2.5)	1 (6.7)	
No	53 (96.4)	39 (97.5)	14 (93.3)	

* Indicates groups with significant differences; ^ PML: progressive multifocal leukoencephalopaty; ^†^ COPD: chronic obstructive pulmonary disease.

**Table 2:** Baseline immunological characteristics of patients with MS treated with anti-CD20 therapies (*n* = 55) and patients treated with OCR (*n* = 40) and RTX (*n* = 15).

Baseline	Total (*n* = 55)	OCR (*n* = 40)	RTX (*n* = 15)	*p*-Value
**Cellular compartment**				
Lymphocytes cells/μL, mean (SD)	1833 (758)	1851 (691)	1789 (927)	0.55
CD4^+^ cells/μL, mean (SD)	834 (338)	895 (344)	662 (263)	0.27
CD8^+^ cells/μL, mean (SD)	413 (274)	407 (227)	432 (393)	0.68
CD19^+^ cells/μL, mean (SD)	188 (151)	170 (114)	210 (225)	0 92
NK cells, mean (SD)	280 (206)	256 (124)	369 (389)	0.95
**Humoral compartment**				
IgG mg/dL, mean (SD)	996 (370)	952 (212)	1069 (542)	0.81
IgA mg/dL, mean (SD)	230 (89)	228 (80)	233 (106)	0.78
IgM mg/dL, mean (SD)	112 (66)	119 (76)	98 (36)	0.87
**B cell compartment**	**(*n* = 28)**	**(*n* = 22)**	**(*n* = 6)**	
Naive cells/μL, mean (SD)	121 (92)	97 (51)	182 (155)	0.82
Unswitched memory cells/μL, mean (SD)	21 (17)	20 (15)	21 (26)	0.39
Switched memory cells/μL, mean (SD)	33 (50)	22 (9)	64 (106)	0.23
Transitional cells/μL, mean (SD)	5 (6)	5 (6)	5 (8)	0.28
Plasmablasts cells/μL, mean (SD)	3 (3)	3 (4)	2 (1)	0.57
CD21^low^ cells/μL, mean (SD)	5 (6)	4 (3)	8 (13)	0.98

**Table 3:** Follow-up of the humoral compartment at the different study time points in patients with MS treated with anti-CD20 therapies (*n* = 55).

	Baseline	6 Months	12 Months	24 Months	*p*-Value
**Total**	***n*** ** = 41**	***n*** ** = 37**	***n*** ** = 33**	***n*** ** = 27**	
Lymphocytes cells × 10^6^/L, mean (SD)	1833(759)	1579 (549)	1685 (638)	1586 (432)	0.1
CD4 cells/µL, mean (SD).	834 (338)	825 (340)	873 (331)	860 (242)	0.8
CD8 cells/µL, mean (SD)	413 (274)	403 (212)	421 (260)	383 (255)	0.1
CD19 cells/µL, mean (SD)	188 (152)	10 (23)	7 (26)	0.2 (1)	0.0000 *
NK cells, mean (SD)	271 (218)	255 (155)	299 (109)	240 (131)	0.8
	***n*** ** = 45**	***n*** ** = 33**	***n*** ** = 28**	***n*** ** = 30**	
IgG mg/dL, mean (SD)	988 (351)	942 (219)	960 (207)	915 (201)	0.4
IgA mg/dL, mean (SD)	231 (90)	243 (98)	255 (106)	211 (89)	0.3
IgM mg/dL, mean (SD)	111 (64)	95 (67)	83 (66)	82 (61)	0.4
**OCR**	***n*** ** = 26**	***n*** ** = 26**	***n*** ** = 26**	***n*** ** = 20**	
Lymphocytes cells × 10^6^/L mean (SD)	1850(690)	1600 (550)	1766 (585)	1578 (336)	0.1
CD4 cells/µL, mean (SD)	895 (344)	861 (325)	909 (335)	836 (229)	0.2
CD8 cells/µL, mean (SD)	406 (226)	413 (403)	431 (215)	385 (218)	0.4
CD19 cells/µL, mean (SD)	170 (114)	3 (4)	5 (20)	1 (1)	0.0001 *
NK cells, mean (SD)	255 (145)	230 (114)	254 (128)	255 (138)	0.5
	***n*** ** = 30**	***n*** ** = 22**	***n*** ** = 21**	***n*** ** = 23**	
IgG mg/dL, mean (SD)	952 (211)	876 (330)	920 (276)	1235 (1653)	0.7
IgA mg/dL, mean (SD)	228 (79)	235 (120)	240 (117)	210 (78)	0.5
IgM mg/dL, mean (SD)	119 (78)	90 (78)	77 (69)	84 (67)	0.4
**RTX**	***n*** ** = 15**	***n*** ** = 11**	***n*** ** = 7**	***n*** ** = 7**	
Lymphocytes cells × 10^6^/L mean (SD)	1790 (926)	1502 (565)	1452 (778)	1643 (685)	0.6
CD4 cells/µL, mean (SD)	661 (263)	1502 (565)	1452 (778)	817 (380)	0.8
CD8 cells/µL, mean (SD)	661 (263)	299 (196)	421 (411)	423 (348)	0.2
CD19 cells/µL, mean (SD)	210 (225)	22 (43)	14 (40)	21 (44)	0.01 *
NK cells, mean (SD)	311 (346)	317 (101)	285 (31)	192 (102)	0.7
	***n*** ** = 15**	***n*** ** = 11**	***n*** ** = 7**	***n*** ** = 7**	
IgG mg/dL, mean (SD)	1069(542)	932 (246)	960 (210)	975 (164)	0.8
IgA mg/dL, mean (SD)	233 (105)	218 (80)	214 (66)	206 (75)	0.5
IgM mg/dL, mean (SD)	233 (105)	84 (47)	75 (38)	70 (42)	0.9

* Indicates groups with significant difference.

**Table 4:** Persistent immunological changes.

	Total (*n* = 55)	OCR (*n* = 40)	RTX (*n* = 15)	*p*-Value
Persistent lymphopenia, *n* (%)	5 (8.9)	3 (7.5)	2 (13.3)	0.51
Persistent hypogammaglobulinemia				
IgG mg/dL, *n* (%)	10 (18.2)	8 (20)	2 (13.3)	0.32
IgM mg/dL, *n* (%)	12 (21.8)	0 (25)	2 (13.3)	0.71
Persistent CD4^+^/CD8^+^ inversion, *n* (%)	3 (6)	3 (7.7)	0	0.74
CD19^+^ = 0 at 6 months, *n* (%)	19 (46.3)	14 (45)	5 (50)	0.82
CD19^+^ = 0 at 12 months, *n* (%)	20 (51.3)	16 (51.6)	4 (50)	0.74

**Table 5:** Infections recorded in multiple sclerosis patients included in the study.

	Total (*n* = 55)	OCR (*n* = 40)	RTX (*n* = 15)	*p*-Value
Total infection, *n* (%)	35 (63.6)	26 (65)	9 (60)	0.76
COVID-19 infection, *n* (%)	22 (63)	18 (45)	4 (26.7)	0.35
Severe, *n* (%)	4 (18)			
Vaccination	52 (94.6)			
Infection prior to vaccination	2 (3.8)			
Non-COVID-19 infection, *n* (%)	13 (37)	8 (20)	5 (33.3)	0.75
Viral upper respiratory tract	3 (23)			
Bacterial pneumonia	1 (7.7)			
Bacterial urinary tract	7 (53)			
Viral gastroenteritis	1 (7.7)			
Bacterial odontogenic infection	1 (7.7)			
**Serious events, ***n* (%)	6 (17.4)	4 (10)	2 (13.3)	0.72
Bacterial pneumonia, *n* (%)	1 (7.7)			
Febrile neutropenia	1 (7.7)			
COVID-19 with severe course, *n* (%)	4 (66)			
COP, *n*	3			

**Table 6:** Infections risk factors.

	Infection (*n* = 30)	No Infection (*n* = 25)	*p*-Value	CI 95%
Age (>50 years)	16 (53.3%)	12 (48%)	0.69	25.99–74.01%
Sex (woman)	16(53.3%)	14 (56%)	0.84	31–79%
Progressive forms, *n* (%)	14 (46.7%)	10 (40%)	0.62	16.48–63.52%
Initial EDSS (mean ± SD)	3.73 ± 2.52	3.8 ± 2.31	0.79	2.363–4.837%
FPT, *n* (%)	21 (70%)	21 (84%)	0.22	61–99%
Lymphopenia, *n* (%)	4 (13.3%)	1 (4%)	0.23	0–15%
IgG hypogammaglobulinemia, *n* (%)	7 (23.3%)	1 (4%)	0.04 *	1.208–2.647%
IgM hypogammaglobulinemia, *n* (%)	4 (13.3%)	5 (20%)	0.51	0.2–24%

* Indicates groups with significant differences. CI 95%: confidence interval 95%.

**Table 7:** Proportion of clinical efficacy parameters observed in patients treated with OCR and RTX.

	Total (*n* = 55)	OCR (*n* = 40)	RTX (*n* = 15)	*p*-Value
EDSS, mean (SD)				
Baseline	3.7 (2.4)	3.2 (2.2)	5.25 (2.4)	0.03 *
Posterior	4.1 (2.5)	3.6 (2.4)	5.5 (2.4)	0.017 *
EDSS, median (interquartile)				
Baseline	4.5 (1.5–6)	2.8 (1.5–5)	6 (2.5–6.5)	
Posterior	4.5 (2–6.5)	2.5 (2–6)	6 (2–7.5)	
Progression EDSS, *n* (%)				0.19
Yes	17 (30.9)	13 (32.5)	4 (26.7)	
No	38 (69.1)	27 (67.5)	11 (73.3)	
New MRI Lesions, *n* (%)				0.07
Yes	5 (9.1)	4 (10)	1 (6.7)	
No	41 (74.5)	33 (82.5)	8 (53)	
Unregistered	9 (16.4)	3 (7.5)	6 (40)	
Relapses, *n* (%)				0.28
Yes	2 (3.6)	1 (2.5)	1 (6.7)	
No	52 (94.5)	39 (97.5)	14 (93.3)	
Activity, *n* (%)				0.18
Yes	6 (10.9)	5 (12.5)	1 (6.7)	
No	49 (89.1)	35 (87.5)	14 (93.3)	
NEDA-3, *n* (%)				0.44
Yes	34 (61.8)	24 (60)	10 (66.7)	
No	21 (38.2)	16 (40)	5 (33.3)	

* Indicates groups with significant differences.

## Data Availability

The data presented in this study are available on request from the corresponding author due to (Data management adhered to the principles of the Spanish Biomedical Research Law 14/2007, ensuring the confidentiality of all personal).

## Supplementary Materials

The following supporting information can be downloaded at https://www.mdpi.com/article/10.3390/diseases13120387/s1: Table S1: Relationship between B-cell lymphocyte subpopulations in the baseline immunophenotype and previous immunological fingerprint (not footprint) therapy.

## Abbreviations

The following abbreviations are used in this manuscript:

MS	Multiple sclerosis
CNS	Central nervous system
RRMS	Relapsing-remitting MS
SPMS	Secondary progressive MS
PPMS	Primary progressive MS
OCR	Ocrelizumab
RTX	Rituximab
DMT	Disease-modifying treatment
MRI	Magnetic resonance imaging
FPT	Fingerprint therapy
EDSS	Expanded Disability Status Scale
ECTRIMS	European Committee for Treatment and Research in Multiple Sclerosis
NEDA-3	No evidence of disease activity-3
COP	Cryptogenic organised pneumonia
PML	Progressive multifocal leukoencephalopathy
