# Supplementary material for: Immunological Monitoring During Anti-CD20 Therapies to Predict Infection Risk and Treatment Response in Multiple Sclerosis Patients
Source: Diseases. 2025 Nov 28;13(12):387. doi: 10.3390/diseases13120387 (PMC12731810; doi:10.3390/diseases13120387)
Supplement: Supplementary file 1 [file diseases-13-00387-s001.zip › diseases-3912845-supplementary.pdf]

Table S1. Relationship between B-cell lymphocyte subpopulations in the baseline immunophenotype and previous immunological fingerprint (not footprint) therapy.

	FPT (*n*=13)	No FPT (*n*=42)	*p*-value (CI)
Naive cells/µL, mean (SD)			
- percentage	67 (14)	62 (19)	0.54 (-14.70 – 25.49)
- absolute	102 (58)	204 (146)	0.14 (-183.16 – -26.36)
Unswitched memory cells/µL, mean (SD)			
- percentage	13 (11)	14 (9)	0.91 (-10.28 – 9.27)
- absolute	17 (13)	34 (24)	0.13 (-42.41 – 6.82)
Switched memory cells/µL, mean (SD)			
- percentage	14 (6)	19 (13)	0.15 (-12.89 – 2.02)
- absolute	22 (18)	32 (50)	0.29 (-152.09 – 52.27)
Plasmablasts cells/µL, mean (SD)			
- percentage	2 (3)	2 (3.2)	0.15 (-0.49 – 3.03)
- absolute	3 (3)	2 (2)	0.84 (-1.92 – 2.34)
$CD21^{low}$ cells/µL, mean (SD)			
- percentage	2 (1)	4 (2)	0.30 (-3.74 – 1.37)
- absolute	3 (1)	3 (6)	0.21 (-20.68 – 5.85)
